# The Yin and Yang of dietary gluten transgressions in real-life scenarios of celiac patients

**DOI:** 10.1186/s12916-020-01535-8

**Published:** 2020-03-11

**Authors:** Aaron Lerner, Torsten Matthias

**Affiliations:** AESKU.KIPP Institute, Mikroforum Ring 2, 55234 Wendelsheim, Germany

**Keywords:** Celiac disease, Gluten, Gluten-free diet, Real-life scenario, Indiscretions, Transgressions, Safety, Dietary adherence, Compliance, Intestinal pathology

## Background

According to the Chinese philosophy, Yin and Yang forces are contradictory, yet complementary, energetic qualities that shape our lives. Positive and negative, dark and bright eternally intermingle on a separate and intertwined spectrum. Food choices, nutrients, or dietary restrictions are not an exception. In this regard, patients with celiac disease (CD) on a gluten-free diet (GFD) live between Yin and Yang, represented by the real-life scenario of bounding back and forth between restricted compliance and temptations to occasional or permanent gluten ingestion. Luca et al. should be congratulated for their real-life scenario study, reporting occasional or voluntary transgression from a GFD in adults with CD [1]. The authors concluded that, despite long-term gluten consumption, no symptomatology nor enteric damage was apparent, suggesting the possibility of acquired gluten intolerance. They substantiated their findings by applying clinical examination, CD-associated serology, duodenal histology, capsule endoscopy, and a validated food-frequency questionnaire. Despite the study’s valuable contribution to the field, numerous controversies and ambiguities still remain concerning CD diagnosis and GFD withdrawal.

From the CD perspective, diagnosis is based on a combination of clinical symptoms, serology, genotyping, and intestinal histology [2]. Presenting symptoms are manifold. The epidemiology is changing toward asymptomatology and hypo-symptomology, and many of the patients present with extra-intestinal manifestations [3]. Shortcomings are associated with using serology for CD diagnosis. Patients should be on a gluten-containing diet and IgA deficiency should be excluded. Antibody sensitivity is lower in a milder intestinal pathology, and even the most frequently used antibody, IgA anti-tissue transglutaminase, can present with multiple false positives and negatives [2, 4]. HLA-DQ2/8 negativity is used to rule out a diagnosis of CD but positivity is only confirmative and cannot be used as a first-line test [2]. In the past, histology was conclusive and necessary for a definitive diagnosis. However, in recent years, ambiguity has arisen due to significant inter-observer disagreement, the patchy histological presentation, shared features with multiple gastrointestinal conditions, and inaccuracy in milder damage [2, 5]. Due to the complexity of the disease and the controversies surrounding its classification and diagnosis, it is apparent that not a single test will make an accurate diagnosis. Therefore, a combined holistic approach to improve and standardize the CD diagnoses is urgently required [2]. Intriguingly, CD nutritional therapy is in no way less ambiguous. Below we discuss some controversies associated with gluten withdrawal.

## Gluten withdrawal: strict cut-off levels

Gluten restriction means complete avoidance of any gluten-containing nutrients as well as any cross-contamination; however, in this aspect, reality does not necessarily match theory. Cross-contamination is pervasive, sometimes reaching 5–50 mg of gluten per day [6]. Gluten content may be substantial even in the less toxic prolamin, namely oat. Hidden gluten is frequent and often unlabeled. Traditionally, the gluten content is expressed in parts per million (corresponding to milligrams per kilogram). However, no agreement exists on safe gluten content threshold levels. Most advocate 20 ppm, but the range can be quite extensive, from 10 to 100 ppm [6]. As is true for many other biological reactions, the cut-off levels are relative and reflect the patient’s sensitivity to the offending agent.

## A tough alley and torrid times for gluten avoiders

Gluten avoiders are currently passing through a tough alley due to the low availability, high cost and inconvenience of gluten-free products as well as their lack of appropriate labeling, low palatability, and compromised texture. Furthermore, gluten abstainers are also at risk of an unbalanced diet and nutritional deficiencies, undergo social peer pressure, persistent symptoms despite a GFD, and an inadequacy of dietary counseling. Gluten avoidance is also made difficult since gluten is found in 70% of processed food products and there is a lack of manufacturing regulations in many countries. Anger, fear, and anxiety following a diagnosis, dissatisfaction with the information provided by professionals, and inaccurate information from food stores, alternative practitioners, family, friends, and other unprofessional or easily accessible sources represent further challenges [7].

Similarly, gluten avoiders are living in contemporary torrid times since the gluten content in wheat is evolutionarily increasing along with gluten consumption and usage, thus affecting the gluten-sensitive population. Simultaneously, there has been an increase in the immunogenicity of CD-related T cell stimulatory epitopes in wheat – a universal food additive. Increased usage of gluten by the processed food industry represents an additional gluten cargo and increased morbidity, co-morbidity, and mortality. The gluten-sensitive population is entrenched between the multiple side effects of gluten and the effects of gluten restriction such as nutritional deficiencies, toxicity, morbidity, mortality, and mental health problems [8].

## Monitoring adherence to GFD

Despite its importance for the health of patients with CD, there are no evidence-based, reasonable, randomized control studies or meta-analysis recommendations for the best way to monitor gluten withdrawal adherence [9]. Clinical symptoms are variable, subjective, unspecific, shared by many gastrointestinal diseases, and geo-epidemiologically dependent. Whilst dietetic reviews aimed at evaluating self-reported GFD adherence questionnaires might be useful to detect gluten transgressions, these need to be adapted to local dietary habits, to local languages, validated to detect the frequency and quantity of gluten intake, and capable of detecting nutritional deficiencies [8]. Nevertheless, no back-to-back comparisons are available to recommend dietary reviews over other means to check gluten avoidance adherence. Strict compliance is challenging since GFD infringement is influenced by psychological, emotional, and social factors, age and way of diagnosis (by screening or clinical suspicion), knowledge of the disease, and eating habits. The contradictory performances of serology [2, 4] and follow-up biopsy [2, 5] as a monitory means of compliance were described above. Recently, two additional, more objective ways to detect dietary gluten transgressions using gluten immunogenic peptide excretion in urine and stool have been described [10]. The fecal and urine excretion of gluten immunogenic peptides represents a new step in objective monitoring of GFD adherence and brings the point-of-care a step closer to achieve the home-based, self-assessment of gluten dietary indiscretions [10].

## Conclusions

A literature review shows us the ambiguities, controversies, and ongoing debates on various aspects of CD, including the fact that its therapy is focused exclusively on GFD, methods to monitor gluten adherence, and the disease’s natural history. Each aspect is challenging, and the question of whether GFD should be permanent and/or for all has re-emerged. Understanding gluten tolerance might lead to a shift in the current paradigms and represent a game-changer in CD therapy, alleviating the complexity of GFD compliance and adherence.

## Data Availability

Not applicable.
